# A Meta-Analysis for Association of Maternal Smoking with Childhood Refractive Error and Amblyopia

**DOI:** 10.1155/2016/8263832

**Published:** 2016-05-09

**Authors:** Li Li, Ya Qi, Wei Shi, Yuan Wang, Wen Liu, Man Hu

**Affiliations:** Department of Ophthalmology, Beijing Children's Hospital, Capital Medical University, National Key Discipline of Pediatrics, Ministry of Education, Beijing 100045, China

## Abstract

*Background*. We aimed to evaluate the association between maternal smoking and the occurrence of childhood refractive error and amblyopia.* Methods*. Relevant articles were identified from PubMed and EMBASE up to May 2015. Combined odds ratio (OR) corresponding with its 95% confidence interval (CI) was calculated to evaluate the influence of maternal smoking on childhood refractive error and amblyopia. The heterogeneity was evaluated with the Chi-square-based *Q* statistic and the *I* ^2^ test. Potential publication bias was finally examined by Egger's test.* Results*. A total of 9 articles were included in this meta-analysis. The pooled OR showed that there was no significant association between maternal smoking and childhood refractive error. However, children whose mother smoked during pregnancy were 1.47 (95% CI: 1.12–1.93) times and 1.43 (95% CI: 1.23-1.66) times more likely to suffer from amblyopia and hyperopia, respectively, compared with children whose mother did not smoke, and the difference was significant. Significant heterogeneity was only found among studies involving the influence of maternal smoking on children's refractive error (*P* < 0.05; *I* ^2^ = 69.9%). No potential publication bias was detected by Egger's test.* Conclusion*. The meta-analysis suggests that maternal smoking is a risk factor for childhood hyperopia and amblyopia.

## 1. Background

Refractive error (including myopia, hyperopia, and astigmatism) and amblyopia are the leading causes of visual impairment worldwide, which are projected to affect at least one-third of the world's population by 2020 [1]. The crude prevalence of hyperopia and myopia in the US, Western Europe, and Australia is estimated to be 5.8%–11.6% and 16.4%–26.6%, respectively [2]. A population-based cohort study within children at the age of 7 years in the UK shows that amblyopia remains as a common problem affecting at least one in 30 children, and the presented data indicated that disadvantaged children are more at risk of hypermetropia [3]. Refractive error and amblyopia have posed serious public health and economic concerns. Thus recognition of any refractive error and amblyopia in children would be a major step for preventing childhood vision loss, while the prevention of refractive error and amblyopia by identifying avoidable or reversible risk factors could have even greater impact on preventing vision loss.

A few of environmental risk factors have been reported to be implicated in development of refractive error or amblyopia, such as family history, near work, and breastfeeding [4–6]. Nowadays, increasing evidence has shown a possible inverse association of parental smoking, especially maternal smoking, with childhood visual impairment. For example, an epidemiologic investigation by Iyer et al. [7] evaluated the relationship of parental smoking with refractive errors in children aged 6–72 months in Singapore and found an association between maternal smoking and lower myopia prevalence among children. In addition, a mean hyperopic shift and less myopia prevalence have been identified to be associated with passive exposure to tobacco smoke from either maternal or paternal during childhood, and a similar relation of children's refractions with either parent smoking during the mother's pregnancy was also observed [8]. Nevertheless, another study from Japan did not discover the association of parental smoking with visual acuity below 0.7 within schoolchildren [9]. Moreover, a cross-sectional study of children from the Multiethnic Pediatric Eye Disease Study and the Baltimore Eye Disease Study suggested that maternal smoking during pregnancy or infancy is associated with a higher risk of having astigmatism [10]. The controversial relationship of maternal smoking with their children's refractive error or amblyopia is urgently needed to be systematically reviewed.

The present meta-analysis extracted information from 9 studies with a total of 42,318 children by application of statistical techniques, hoping to draw a convincible conclusion about the effect of maternal smoking on the occurrence of childhood refractive error and amblyopia.

## 2. Materials and Methods

### 2.1. Data Sources and Search Strategy

Relevant articles were identified* via* a systematic search through PubMed (http://www.ncbi.nlm.nih.gov/pubmed/) and EMBASE (http://www.embase.com) up to May 2015. Search strategy was applied as follows: [(refractive error OR (nearsightedness OR myopia) OR (farsightedness or hyperopia) OR astigmatism OR amblyopia) AND (smoking OR smoke)]. Reference lists from the included articles were also scanned for more relevant studies.

### 2.2. Inclusion and Exclusion Criteria

Articles were independently selected by two investigators. Studies were included in this meta-analysis if they met the following criteria: (i) the study included children suffering from refractive error (including myopia, hyperopia, and astigmatism) and/or amblyopia; (ii) children with normal vision were regarded as control; (iii) the odds ratio (OR) was provided or could be calculated for accessing association of subject refractive error or amblyopia with maternal smoking status during pregnancy; (iv) the study was published in English.

Studies were excluded if they were not original research but reports, reviews, letters, or comments.

### 2.3. Data Extraction and Quality Assessment

The following information was independently extracted by two investigators by using a devised data extraction form from the eligible studies: the first author, publication year, study location, study time, age of the children, and so forth. Results were compared and discrepancies were resolved by discussing within our research team until a consensus was reached.

### 2.4. Statistical Analysis

The statistical analysis was performed using RevMan 3.11. The heterogeneity among the included studies was evaluated with the Chi-square-based *Q* statistic (*χ* ^2^) and the *I* ^2^ test [11]. If there was no significant heterogeneity (*P* > 0.05 and *I* ^2^ < 50%), the fixed effect model was chosen for meta-analysis; otherwise, the random effects model would be used [12]. Combined odds ratio (OR) corresponding with its 95% confidence interval (CI) was calculated to assess the influence of maternal smoking on children's visual acuity. *P* ≤ 0.05 was considered the existence of statistical differences.

Sensitivity analysis was further conducted to detect the influence of each study on the overall effect by omitting one study at a time [13]. Potential publication bias was finally examined by the visual inspection of Egger's test [14].

## 3. Results

### 3.1. Search Results

**Figure 1:**
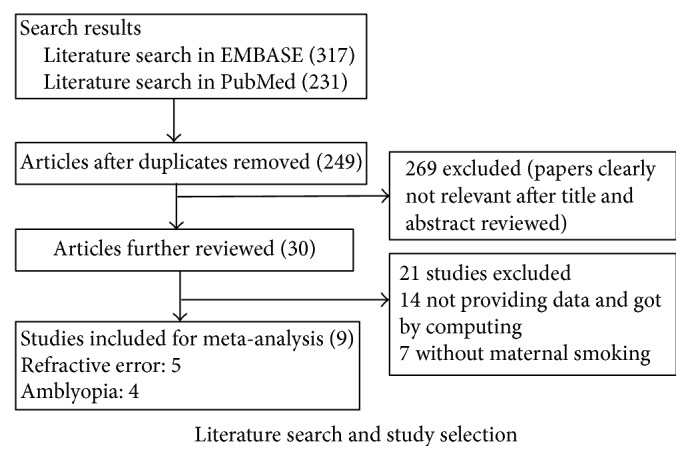
Flowchart of studies search and selection.

Based on the search strategy, 548 potentially relevant articles (317 from EMBASE and 231 from PubMed) were finally identified. Among these articles, 299 were excluded for duplicate publication. Subsequently, 269 articles were excluded after reviewing the titles and abstracts, and 30 studies were reserved for more detailed evaluation. The full texts of the remaining articles were studied and 21 were further excluded, including 14 studies without sufficient data such as the style of refractive error (including myopia, hyperopia, and astigmatism and amblyopia) and OR with its corresponding 95% CI for maternal smoking with childhood refractive error and amblyopia and 7 studies without maternal smoking. Finally, 9 articles were retrieved for the present meta-analysis [3, 7, 8, 10, 15–19]. Flowchart for searching process is shown in Figure 1.

### 3.2. Study Characteristics

The main characteristics of eligible studies were listed in Table 1. These articles included a total of 42,318 children, among which 3,282 were suffering from refractive error and 318 with amblyopia. The ages of children involved in these studies ranged from 6 to 72 months. These 9 studies were published from 2006 to 2013 and were mainly from Singapore, the United States, Australia, and England. Five studies [7, 8, 10, 15, 19] involve the influence of maternal smoking on children's refractive error. Specifically, there were 3 [7, 8, 10], 2 [10, 19], and 1 [15] studies focused on myopia, hyperopia, and astigmatism, respectively. Four studies [3, 16–18] involved the influence of maternal smoking on amblyopia.

### 3.3. Meta-Analysis for Influence of Maternal Smoking on Children's Refractive Error

**Figure 2:**
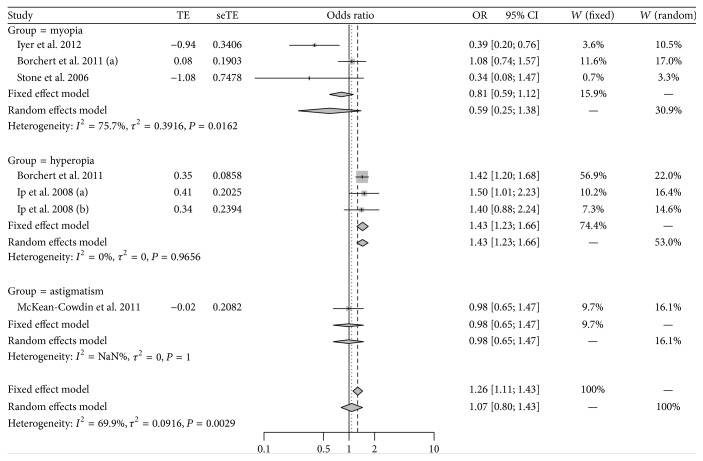
Meta-analysis for association of maternal smoking with childhood refractive error.

**Figure 3:**
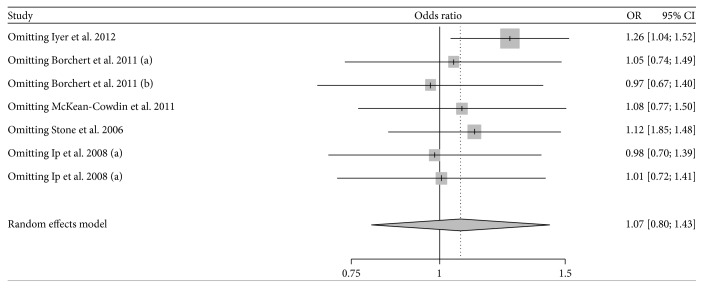
Sensitivity analyses for the influence of each study involving refractive error on the overall effect.

As shown in Figure 2, significant heterogeneity was found among individual studies involving the influence of maternal smoking on children's refractive error (*P* < 0.05; *I* ^2^ = 69.9%), and then the random effect model was chosen for meta-analysis. Children whose mother smoked during pregnancy were 1.07 times more likely to have refractive error than children whose mother did not smoke, while the result was not statistically significant (95% CI: 0.80–1.43). No potential publication bias was detected by Egger's test (*t* = 2.48; *P* = 0.06). Sensitivity analysis showed that the pooled result was altered after omitting the study by Iyer et al. [7] and became statistically significant (OR = 1.26; 95% CI: 1.04–1.52; Figure 3). When the meta-analysis was stratified by different types of refractive error, the occurrence of hyperopia was found to be significantly different between children whose mothers smoked during pregnancy and those whose mothers did not smoke (OR = 1.43; 95% CI: 1.23–1.66), while no statistical difference was found in myopia (OR = 0.59; 95% CI: 0.25–1.38) or astigmatism (OR = 0.98; 95% CI: 0.65–1.47) (Figure 2).

### 3.4. Meta-Analysis for Influence of Maternal Smoking on Children's Amblyopia

**Figure 4:**
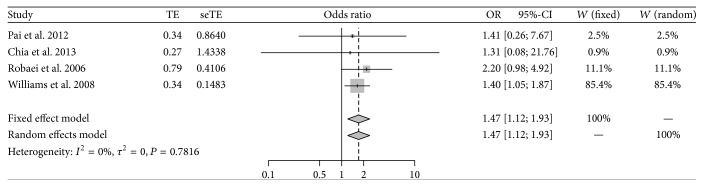
Meta-analysis for association of maternal smoking with childhood amblyopia.

**Figure 5:**
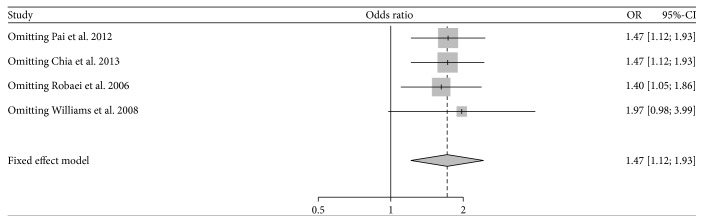
Sensitivity analyses for the influence of each study involving amblyopia on the overall effect.

As shown in Figure 4, no significant heterogeneity was found among individual studies involving the influence of maternal smoking on children's amblyopia (*P* = 0.782; *I* ^2^ = 0.00%); therefore, the fixed effect model was chosen for meta-analysis. Based on the pooled OR, children whose mother smoked during pregnancy were 1.47 times more likely to have amblyopia than those whose mother did not smoke, and the result was statistically significant (95% CI: 1.12–1.93). No potential publication bias was detected by Egger's test (*t* = 0.57; *P* = 0.624). Sensitivity analysis showed that the pooled result was slightly changed (OR = 1.97; 95% CI: 0.98–3.99) but became no longer statistically significant (*P* = 0.06) after omitting the study by Williams et al. [3] (Figure 5).

## 4. Discussion

To the best of our knowledge, this is the first meta-analysis to evaluate the influence of maternal smoking on the children's refractive error and amblyopia. Our aim was to draw a convincible conclusion regarding this influence. This meta-analysis involved 9 articles and included a total of 42,318 children among which 3,282 were suffering from refractive error and 318 with amblyopia. Results demonstrated that children whose mother smoked during pregnancy were 1.47 times more likely to suffer from amblyopia compared with children whose mother did not smoke. No statistical difference was found in refractive error between the two groups of children, but the occurrence of hyperopia was found to be significantly higher in children whose mother smoked during pregnancy than those whose mother did not smoke when the studies were stratified by different type of refractive error.

Potential factors related to the refractive error and amblyopia, as well as the underlying mechanisms which may be responsible for the failure of emmetropization, are all not well understood. Recent studies have hypothesized that nicotinic acetylcholine receptors may be essential in refractive development, and animal models suggest that drugs blocking nicotinic acetylcholine receptors are associated with the development of refractive error [20, 21]. Nicotine, as one of the important active constituents of cigarette smoke, may elicit a wide range of complex and sometimes conflicting biological effects by activating nicotinic acetylcholine receptors [22]. The present study demonstrated an association of maternal smoking with childhood hyperopia and amblyopia, which supports the notion that the eye growth may be regulated by nicotinic acetylcholine receptors* via* antagonizing to the muscarinic acetylcholine receptors which promote axial elongation of the eye [8]. Nevertheless, nicotinic antagonists experimentally inhibit the myopia in animal model, making it unlikely that nicotinic agonists consisting in tobacco do the same [21]. Therefore, the biological explanation for the association of childhood hyperopia and amblyopia with maternal smoking remains speculative.

In our analysis, heterogeneity was discovered among individual studies involving the influence of maternal smoking on children's refractive error. The 9 articles qualified in our meta-analysis were studied in different countries, and the prevalence of women who smoke is varied. On the other hand, race or ethnicity was reported to be associated with refractive errors and astigmatism [10, 23]. Thus, the study location and race or ethnicity may be the main source of heterogeneity. The different definitions may be one explanation for the differing results from studies and the heterogeneity among studies involving amblyopia in the present study. Myopia is generally defined as an SER of −0.50 D or less, and hypermetropia is generally defined as an SER of +0.50 D or greater. Nevertheless, amblyopia may be defined using a threshold level of cylindrical refractive error in the right or left eye of ≥ 1.50 D expressed in positive correcting cylinder form, using MEPEDS criteria and divided into unilateral and bilateral subtypes, or with VA < 20/30 in the worse eye and VA < 20/40 in both eyes and so forth.

We have to acknowledge that the present meta-analysis has several limitations. First of all, a relatively small number of articles were enrolled in this meta-analysis, which indicated that the results should be concluded cautiously. Secondly, the pooled result was reversed by sensitivity analysis, suggesting that more relevant studies are needed and updated meta-analyses should be conducted. Thirdly, meta-analysis, as a retrospective research tool, is subject to methodological deficiencies, such as lack of appropriate control group and poor representative of population. Finally, our results may be confounded by social class, diet, alcohol intake, and prematurity and some confounding effects, such as socioeconomic factors, could perhaps account for our findings that parental smoking was associated with childhood amblyopia. This information, however, is limited in the eligible studies and has not been analyzed.

Despite the above limitations, we concluded that maternal smoking is a risk factor for childhood hyperopia and amblyopia. This is important because it represents a modifiable factor that can be targeted with education. However, updated meta-analyses including more studies are needed to confirm our results.

## Figures and Tables

**Table 1:** Characteristics of the included studies.

Author	Publication year	Study location	Studying time	Age	Style	N1	N2	Cycloplegia	Adjustments	OR, 95% CI	Definitions
Iyer et al. [7]	2012	Singapore	NA	40.5 (6–72) m	Refractive error: myopia	137	2502	Yes	Adjusted for age, gender, total family income, father's and mother's education, height, parental myopia, reading words or picture books, and total time spent outdoors	0.39 (0.20–0.76)	The definition of myopia was SER of at least −0.5 D

Borchert et al. [10]	2011	USA	NA	6 to 72 m	Refractive error: myopia	378	9515	Yes	No	1.08 (0.74–1.56)	Myopia was defined as SER error ≤−1.00 D
			NA	6 to 72 m	Refractive error: hyperopia	1788	6781	Yes	No	1.42 (1.20–1.68)	Hyperopia was defined as SER error ≥+2.00 D

McKean-Cowdin et al. [15]	2011	USA	NA	6 to 72 m	Refractive error: astigmatism	859	7720	Yes	Adjusted for age, race, and spherical equivalent of the right eye	0.98 (0.65–1.47)	Astigmatism was defined using a threshold level of cylindrical refractive error in the right or left eye of ≥1.50 D expressed in positive correcting cylinder form

Stone et al. [8]	2006	USA	NA	8.7 ± 4.4 y	Refractive error: myopia	63	279	Yes	No	0.34 (0.08–1.50)	Myopia was defined as a spherical equivalent refraction of ≤−0.5 D for the mean of both eyes

Pai et al. [16]	2012	Australia	2007–2009	6–72 m	Amblyopia	27	1395	Yes	Adjustments for age, gender, ethnicity, and SER	1.41 (0.26–7.69)	Amblyopia was defined using the MEPEDS criteria and divided into unilateral and bilateral subtypes

Chia et al. [17]	2013	Singapore	2006–2009	30–72 m	Amblyopia	20	1662	Yes	No	1.31 (0.08–22.08)	Unilateral amblyopia was defined as a 2-line difference between eyes with VA < 20/30 in the worse eye; bilateral amblyopia was defined as VA in both eyes < 20/40

Robaei et al. [18]	2006	Australia	2003-2004	6 years old	Amblyopia	32	1733	Yes	No	2.20 (1.00–5.00)	Amblyopia was initially defined as corrected VA less than 0.3 logMAR units in the affected eye not attributable to any underlying structural abnormality of the eye or visual pathway plus a difference of at least 2 logMAR lines between the 2 eyes

Williams et al. [3]	2008	UK	1998–2000	NA	Amblyopia	239	6696	No	No	1.40 (1.04–1.86)	Amblyopia was defined as those with a history of patching treatment and/or with an interocular difference in best acuity for each eye of 0.2 logMAR units where the worst-seeing eye had a best acuity of worse than 0.3 logMAR, and the eye looked normal on dilated funduscopy

Ip et al. [19]	2008	Australia	NA	6 years old	Refractive error: Hyperopia1	35	153	Yes	No	1.50 (1.04–2.30)	Hyperopia was defined as an SER of +0.50 D or greater and was stratified as mild or moderate
			NA	12 years old	Refractive error: Hyperopia2	22	282	Yes	No	1.40 (0.90–2.30)	Myopia was defined as an SER of −0.50 D or less

m: months; y: years; N1: with refractive error or amblyopia; N2: without refractive error and amblyopia; EVA: Electronic Visual Acuity; SPEDS: the Sydney Paediatric Eye Disease Study; SER: spherical equivalent refraction; MEPEDS: Multiethnic Pediatric Eye Disease Study; D: diopters; VA: visual acuity.
